# Supplementary material for: Increased elastase sensitivity and decreased intramolecular interactions in the more transmissible 501Y.V1 and 501Y.V2 SARS-CoV-2 variants’ spike protein–an in silico analysis
Source: PLoS One. 2021 May 26;16(5):e0251426. doi: 10.1371/journal.pone.0251426 (PMC8153447; doi:10.1371/journal.pone.0251426)
Supplement: Attachment — Submitted filename: Response to Reviewers.docx [file pone.0251426.s003.docx]

**Review Comments**

**Reviewer #1:** The authors used multiple computational methods like MOE analysis, ExPASy, and sequence alignment to evaluate the influence of 501Y.V1 and 501Y.V2 mutants of S protein on SARS-Cov-2 transmissibility. The underlying mechanism, as proposed by the authors, is that the mutations impair the intramolecular interactions of S protein and thus promote the transition from its closed conformation to the opened one, increasing the probability of binding to ACE2. However, such a hypothesis was made simply on the interaction analysis of the static structure of S protein. The structural dynamics, which might be achieved by other experimental and/or computational methods, e.g., molecular dynamics simulation, was not presented, giving no solid evidence to conduct the conclusions. Thus, I don’t think the article in the present form is eligible for publication as a research article.

Reply: In order to address this comment, we modified the title of the article to clearly indicate that this is – an *in silico* analysis, reflecting also the opinion of reviewer #2.

**Reviewer #2:** The authors, Pokhrel et al., describe a decreased intramolecular interaction of the homotrimer within the spike protein of the more transmissible SARS-CoV-2 lineages in silico. Furthermore, the authors found additional neutrophil elastase (NE) cleavage sites, which are introduced in the novel SARS-CoV-2 lineages. Although the manuscript lack of experiments, the findings are of interest. The following concerns should be addressed.

Major concerns:

1. In general, the abstract is missing the findings of neutrophil elastase (NE).

Reply: This is now added (relates also to comment 2).

2. Small aliphatic residues, in particular valine and alanine, and to a lesser extent threonine and isoleucine, are preferred by NE at P1 Kasperkiewicz et al., 2014 (10.1073/pnas.1318548111) and Schilling and Overall, 2008 (10.1038/nbt1408). These manuscripts should be cited and comparted with obtained data from the prediction tool of ExPASy.

Reply: We thank the reviewer, and these references are now added.

Notably, the mutation of B1.1.7 lineage shows an amino acid exchange of threonine (T) to isoleucine (I) and the B.1.351 lineage, A701V. Why would isoleucine induce a new cleave site compared to threonine or alanine to valine when both are accepted for NE in P1 position?

Reply: We now cite reports that explain the above and have added this to the manuscript:

a. Fu et al. (2018) and Schilling & Overall (2008) demonstrate that I in the P1 site and not the T is preferred by elastase – which explains B1.1.7 T716I analysis. The publications provided by the reviewer indicate that NE can also cleave when T is in the P1 site. Therefore, preference of T or I at the proteolysis site may also depend on what other residues are present around it.
b. Human neutrophil elastase seems to prefer V at P1 position over A, thus explaining the potential gain in B.1.351 A701V (Fu et al. 2018 and Schilling & Overall 2008 in the manuscript).

3. The description of the source of elastase (human or porcine; pancreas or neutrophil derived elastase) for prediction is missing, for instance human elastase shows a slightly different preference for amino acids.

Reply: We agree that this is important to highlight; ExPASy utilizes both human and murine neutrophil elastase to make predictions, and both enzymes share similar catalytic activity (Wiesner et al. (2005): https://doi.org/10.1016/j.febslet.2005.08.056); we focused on the specificity of human elastase (now indicated).

Minor concerns:

1. Why 2 titles?

Reply: We removed the subtitle and added, ‘501Y.V1 and 501Y.V2’ and ‘an *in silico* analysis’ to the title (the latter per the comment of reviewer #1.)

2. One letter amino acid code instead of three letter code should be used constantly. Otherwise it is confusing. For instance, table 1 both are used.

Reply: Now corrected all to be consistent to three letter abbreviations, the more commonly used abbreviations.

3. Table 1c: The manuscript regarding affinity of S protein (B.1.1.7 vs. B.1.531) to ACE2 need to be mentioned/discussed https://doi.org/10.1101/2021.02.22.432359. The authors performed “microscale thermophoresis” for binding studies. Or Collier et al., 2021 (<https://www.citiid.cam.ac.uk/wp-content/uploads/2021/02/POST-SUBMISSION_vaccine-DCv2-2.pdf>)

Reply: We have added discussion of this point in the manuscript [refs 31, 32]

4. Page 2, introduction, 1. Paragraph: The S protein has 1273 aa, 3,540 substitutions found (bioRxiv. 2021:2021.01.04.425340), how can it be that the S protein is 4 times mutated as mentioned in the text?

Reply: Corrected now to ‘almost 3 times per position’.

5. Page 2, introduction, 2. Paragraph: B.1.1.7 variant transmissible 40-70%, seems high. New publication should be cited. To my knowledge it is rather around 35%.

Reply: An increased fitness (transmissibility) of the variants has been reported in various reports over that of the Wuhan variant and, more recently, over whatever variants that was circulating at the time (*e.g.* D614G variant). This may explain the difference in the numbers that we cited vs. those that the reviewer cites. We now explain this point and provide a recent reference that addresses it (van Dorp et al. (2021): doi: 10.1101/2021.03.29.21254233).

6. Page 2, results, 3. Paragraph: Activated neutrophils, in addition to NE, these cells also secret proteinase 3, cathepsin G, NSP-4.

Reply: We agree with the reviewer and have added the following clarification in the discussion: ‘In addition to neutrophil elastase, neutrophils also secrete proteinase 3, cathepsin G and NSP-4. Proteinase 3 has a similar primary sequence specificity to neutrophil elastase [23]. It is less likely that cathepsin G participates in S1 proteolysis, since this protease prefers aromatic or positively charged amino acids in P1 [41] and the mutations did not generate such sites. Similarly, NSP4 has a strong preference for arginine at P1 and therefore could augment furin cleavage sites [42] provided that they were not mutated in the given variant.’ (References are numbered as in the manuscript).

7. Figure 2. UK variant or B.1.1.7 and South African variant or B.1.351 should be written in the figure, above a) and b). Additionally, arrows to make it clear at which position the NE cleavage occurs.

Reply: The figure is now modified per the reviewer’s suggestion. You did not add arrows

8. Figure 4. Needs a higher resolution, in this form figure 4 is not readable.

Reply: Now provided.

9. NE and S protein, Mustafa et al. 2021 (doi.org/10.1021/acsomega.1c00363) should be discussed.

Reply: This study is now discussed. We have added the following text: ‘Neutrophil elastase appears to cleave near the RRAR (amino acids Arg682 to Arg685) of the furin cleavage site [42].’.

6. PLOS authors have the option to publish the peer review history of their article ([what does this mean?](https://journals.plos.org/plosone/s/editorial-and-peer-review-process#loc-peer-review-history)). If published, this will include your full peer review and any attached files.
Reply: We agree to publish the history of our article.


[NOTE: If reviewer comments were submitted as an attachment file, they will be attached to this email and accessible via the submission site. Please log into your account, locate the manuscript record, and check for the action link "View Attachments". If this link does not appear, there are no attachment files.]

While revising your submission, please upload your figure files to the Preflight Analysis and Conversion Engine (PACE) digital diagnostic tool, <https://pacev2.apexcovantage.com/>. PACE helps ensure that figures meet PLOS requirements. To use PACE, you must first register as a user. Registration is free. Then, login and navigate to the UPLOAD tab, where you will find detailed instructions on how to use the tool. If you encounter any issues or have any questions when using PACE, please email PLOS at [figures@plos.org](mailto:figures@plos.org). Please note that Supporting Information files do not need this step.
